# Identification of rice leaf diseases and deficiency disorders using a novel DeepBatch technique

**DOI:** 10.1515/biol-2022-0689

**Published:** 2023-08-28

**Authors:** Mayuri Sharma, Chandan Jyoti Kumar, Jyotismita Talukdar, Thipendra Pal Singh, Gaurav Dhiman, Ashutosh Sharma

**Affiliations:** Department of CSE, Assam Royal Global University, Guwahati, Assam, India; Department of CS & IT, Cotton University, Guwahati, Assam, India; Department of CSE, Tezpur University, Tezpur, India; School of Computer Science Engineering & Technology, Bennett University, Greater Noida, India; Department of Electrical and Computer Engineering, Lebanese American University, Byblos, Lebanon; Department of Computer Science and Engineering, University Centre for Research and Development, Chandigarh University, Gharuan, 140413, Mohali, India; Department of Computer Science and Engineering, Graphic Era Deemed to be University, Dehradun, 248002, India; Division of Research and Development, Lovely Professional University, Phagwara, India; Chitkara University Institute of Engineering and Technology, Chitkara University, Rajpura, Punjab, India; Department of Computer Science, Government Bikram College of Commerce, Patiala, India; School of Computer Science, University of Petroleum and Energy Studies, Dehradun, India

**Keywords:** rice, plant disease, classification, diagnosis, DeepBatch

## Abstract

Rice is one of the most widely consumed foods all over the world. Various diseases and deficiency disorders impact the rice crop’s growth, thereby hampering the rice yield. Therefore, proper crop monitoring is very important for the early diagnosis of diseases or deficiency disorders. Diagnosis of diseases and disorders requires specialized manpower, which is not scalable and accessible to all farmers. To address this issue, machine learning and deep learning (DL)-driven automated systems are designed, which may help the farmers in diagnosing disease/deficiency disorders in crops so that proper care can be taken on time. Various studies have used transfer learning (TL) models in the recent past. In recent studies, further improvement in rice disease and deficiency disorder diagnosis system performance is achieved by performing the ensemble of various TL models. However, in all these DL-based studies, the segmentation of the region of interest is not done beforehand and the infected-region extraction is left for the DL model to handle automatically. Therefore, this article proposes a novel framework for the diagnosis of rice-infected leaves based on DL-based segmentation with bitwise logical AND operation and DL-based classification. The rice diseases covered in this study are bacterial leaf blight, brown spot, and leaf smut. The rice nutrient deficiencies like nitrogen (N), phosphorous (P), and potassium (K) were also included. The results of the experiment conducted on these datasets showed that the performance of DeepBatch was significantly improved as compared to the conventional technique.

## 1 Introduction

**Figure 1:**
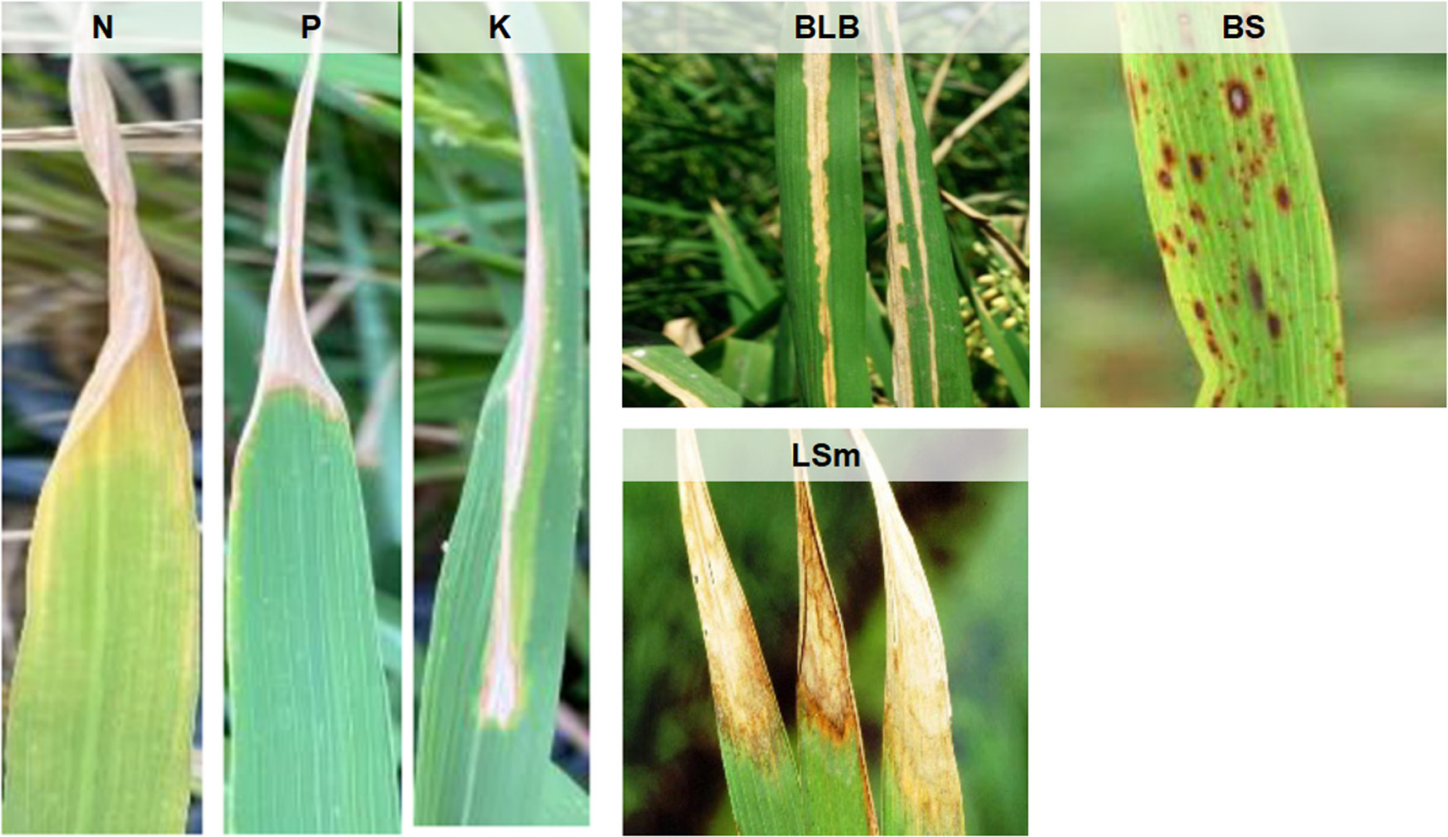
Some common diseases and nutrient deficiency disorders found in rice plants.

The world’s population is growing day by day and will tend to increase at higher rates in the near future. The world's population has increased from 2.5 to 7.7 billion between 1950 and 2019. According to the 2019 UN report, it is predicted that the world’s population would increase by 10, 26, and 42% in the periods of 2019–2030, 2019–2050, and 2019–2100, respectively. The growing population will lead to higher food consumption which in turn will increase food demand and require supply. Asia, the world’s largest continent, relies heavily on rice as its staple food. Between 1961 and 2019, the world’s rice production increased from 215 to 755 million tonnes. Global rice yield increased from 1.86 to 4.66 tonnes per hectare between 1961 and 2019 [1,2,3]. As the population has grown, so have the rice production and its yield. In this manner, the rate of growth in rice yield and production must be sustained over the subsequent decades so that poverty, hunger, undernourishment, food security, etc., do not reach the critical point of failure. However, disease and nutrient deficiency disorders in rice plants act as hindrances to rice growth and yield. Numerous catastrophic diseases that affect rice plants are caused by fungi, bacteria, viruses, and lack of nutrients. Some of the common diseases and nutrient deficiencies in rice plants are shown in Figure 1. Bacterial leaf blight (BLB) disease alone can cause up to 50% yield losses worldwide. Rice yields can also be significantly impacted by some diseases like brown spot (BS) and leaf smut (LSm). One of the most common diseases affecting rice is BS, caused by *Drechsleraoryzae*. BS lesions are often curved, brown in color, and surrounded by a yellow rim. There had previously been evidence of up to 90% of yield losses, which contributed to the Bengal Famine during the 1940s. In Asia, the disease causes yield reductions between 6 and 90% [4,5]. Another disease found in the rice plant is Lsm, which is caused by *Entylomaoryzae*. This disease causes small, darkened patches on the undersides of the leaves. Since it does not significantly reduce the yield, it is regarded as a minor disease. However, severe LSm accelerates the drying of the leaves prematurely, so this disease cannot be ignored. In addition, rice plants also suffer from disorders due to deficiencies of macro/micronutrients like nitrogen (N), phosphorous (P), potassium (K), calcium (Ca), and magnesium (Mg). Rice crops with low NPK levels are investigated more. Lack of P and K in rice crops causes stunted root growth, drought, restricts photosynthesis and makes the plant more susceptible to diseases.

N deficiency reduces the protein and chlorophyll content of rice, lowers yields, and slows the growth of crops [6]. Furthermore, it is now simpler to access and analyze data on these specific contaminations. This is because there is already a lot of data on these common contaminations found in rice crops [1,7,8]. This study, therefore, provides a diagnosis of the three common diseases (BLB, BS, and LSm) and three common nutrient deficiencies (N, P, and K) found in rice plants.

A diagnostic method is necessary for the early detection of rice disease, which can reduce the likelihood of crop loss and increase productivity. The conventional intervention for controlling rice diseases like chemical control, biological control, etc., must be carried out before the plants become adversely affected, which can be accomplished by carefully monitoring the crop. Therefore, there is a need for effective solutions, which can be used to preserve rice growth and yield from diseases and pests [5,8,9]. One such solution is precision agriculture, which essentially involves the application of advanced technology, software tools, and intelligent embedded devices in agriculture. To better regulate and enhance farming practices, the data collected from the deployed sensors are processed and analyzed using machine learning (ML) and deep learning (DL) algorithms. Recently, ML has emerged as a useful technique for the early diagnosis of rice diseases and deficiency disorders. Most research on rice disease diagnosis using RGB images of rice leaves focuses either on classifying disease classes or segmenting the region of interest (ROI). With enormous data, rice disease diagnosis can be done without physical examination of defects in all plants in a dispersed farm. The implementation of ML with IoT tools spread interest in the research for the development of computer systems to assist farmers. For the analysis of rice diseases, computer systems using technologies, viz. spectroscopy based on reflectance, electrical, Fourier transform, and chlorophyll fluorescence, have already proven useful for rice disease diagnosis. Traditional ML models like support vector machines (SVMs), self-organizing models, decision trees, neural networks (NN), clustering techniques, and thresholding are used for infected area segmentation, rice disease classification, and disease recognition. The diagnosis accuracies of these models have been improved by the implementation of DL and ensemble learning models [10,11,12].

In recent days, transfer learning (TL) models are becoming more prominent [13,14,15,16,17]. When tested on field data of 24 rice diseases, VGG16 and GoogleNet accurately predicted the diseases with accuracy rates of 91–92% [18]. TL models such as InceptionV3, EfficientNet, and VGG19 made an accurate prediction in the range of 94–96.08% on a smaller number of rice diseases [16,18,19,20,21]. The use of Mask RCNN on six on-field disease images improved the rice disease diagnostic accuracy of Faster RCNN (70.96%), which was further boosted to 79.19% by Yolov3. Faster RCNN made an 85.4% accurate prediction using only 3 on-field rice diseases, while Mask RCNN boosted this prediction accuracy by 2.05% [22,23,24,25]. With the help of CNN’s super-resolution and classification layers, a dataset on rice disease that was compiled through online and field research showed a disease prediction accuracy of 93.257% [26]. Diagnostic accuracy on public or on-field datasets, or a mix of both, ranged from 94.07 to 98.63% when the inception module was included in TL models like DenseNet and MobileNet. DNN optimized by the Jaya optimization algorithm (DNN_JOA) and the big bang–big crunch–CNN are some more hybrid models [12,27,28]. The recognition accuracy of TL models would increase if high-quality data are provided. Therefore, enhancement of the rice leaf disease image data based on GAN and super-resolution is also given importance to obtain high-quality data [25,29]. The development of ML approaches led to the use of ensemble learning for crop disease diagnosis prediction, locating nutrients, and determining crop yield [8,30,31]. The best result for diagnosing six rice diseases in rice plants using ensemble voting applied to DL-based models was 91%. SegNet segmentation followed by RideSpider Water Wave-based Deep Recurrent Neural Network (Deep RNN) was found to detect rice diseases with the highest accuracy of 90.5% [28].

The commonly used detection method for nutrient deficiency disorder in rice leaves is the regression model. Various color and vegetation indices were computed using color channels. The nitrogen status of a particular rice cultivar was assessed using the Kawashima index where the coefficient of determination between the derived indices and the reference soil plant analysis development (SPAD) value was 0.56. It is also crucial to remember that in a few investigations incorporating nitrogen, chlorophyll SPAD values were the objective variable rather than nutritional content [32,33]. The normalized difference index, canopy cover, and raw values from the red channel were used as inputs in the model for the estimation of the nitrogen state in rice. The coefficient of determination between the proposed model and the nitrogen accumulation ranged from 0.75 to 0.83 [34]. In order to estimate the nitrogen content of a single rice variety, normalized green and red channels were obtained from RGB images and sent as inputs to a quadratic model, with an average prediction accuracy of 75% [35]. When image features including G−R, G + R, and canopy cover were employed separately to build exponential models for nitrogen content in two rice varieties, the coefficient of determination was found in the range of 0.90–0.95 [36]. Eleven different nutrient deficiency disorders in rice plant leaves were detected using pre-trained CNN models, of which NasNet-large and DenseNet121 had the highest detection accuracy of 97.44 and 96.25%, respectively [37]. Pre-trained CNN models were merged with SVM to predict four levels of N insufficiency in rice with the best result being 99.84% [38].With the best prediction result of 100%, ensemble averaging of TL models diagnosed the nutritional deficiencies in rice plants. The same work was tested using a weighted-average ensemble of TL models, which gave an accuracy of 98.33% [8,39].

**Figure 2:**
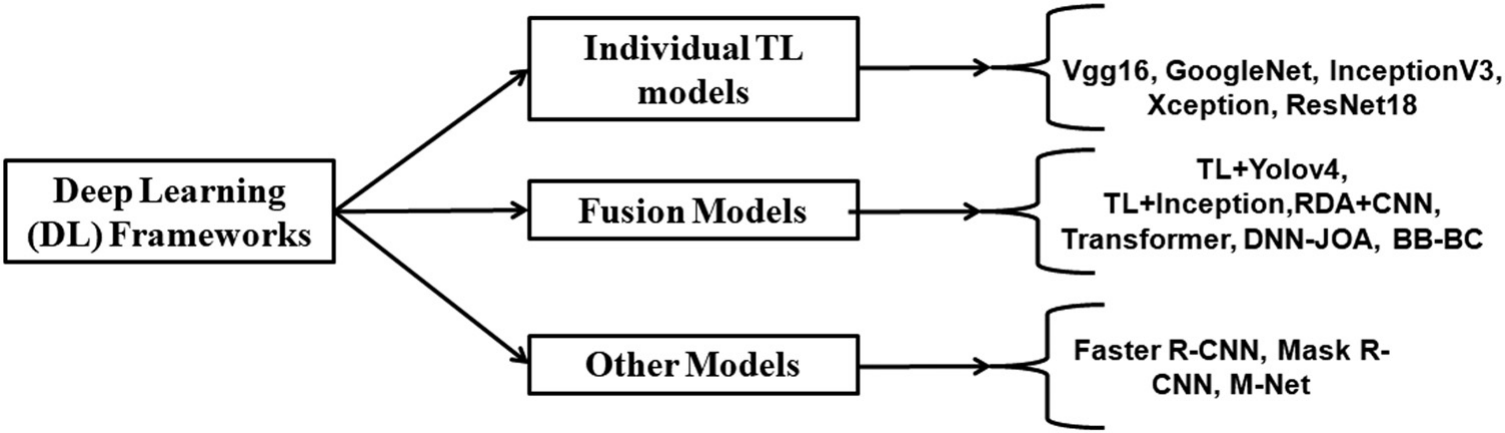
DL framework applied in rice disease classification using image data.

Figure 2 shows the various DL frameworks used in rice disease/deficiency disorder classification using image data. From the literature, it is obvious that DL-based algorithms outperform traditional ML algorithms in the case of rice disease diagnostic systems.

In our previous studies [5,8,40], we built diagnostic systems with various ML/DL techniques. We used various feature selection techniques as well as the ensembling of various classifiers to increase the performance of the system designed. However, effective segmentation may further improve the performance of such DL-based models. Therefore, we propose a DeepBatch (DL-based) diagnostic system where infected rice regions will be extracted and will be fetched to a DL-based classifier.

The main goal of this work is to assist the agricultural expert with a second opinion for the analysis of rice images infected due to the presence of pathogens and nutrient deficiencies to improve the diagnosis. Effective segmentation of the contaminated area in rice leaves can improve the performance of the classification system while constructing a rice disease and deficiency disorder diagnosis system. The current method for designing the diagnostic system has a segmentation module which is based on dilated convolutions and a pre-trained model followed by a TL-based classification module.

## 2 Methodology

**Figure 3:**
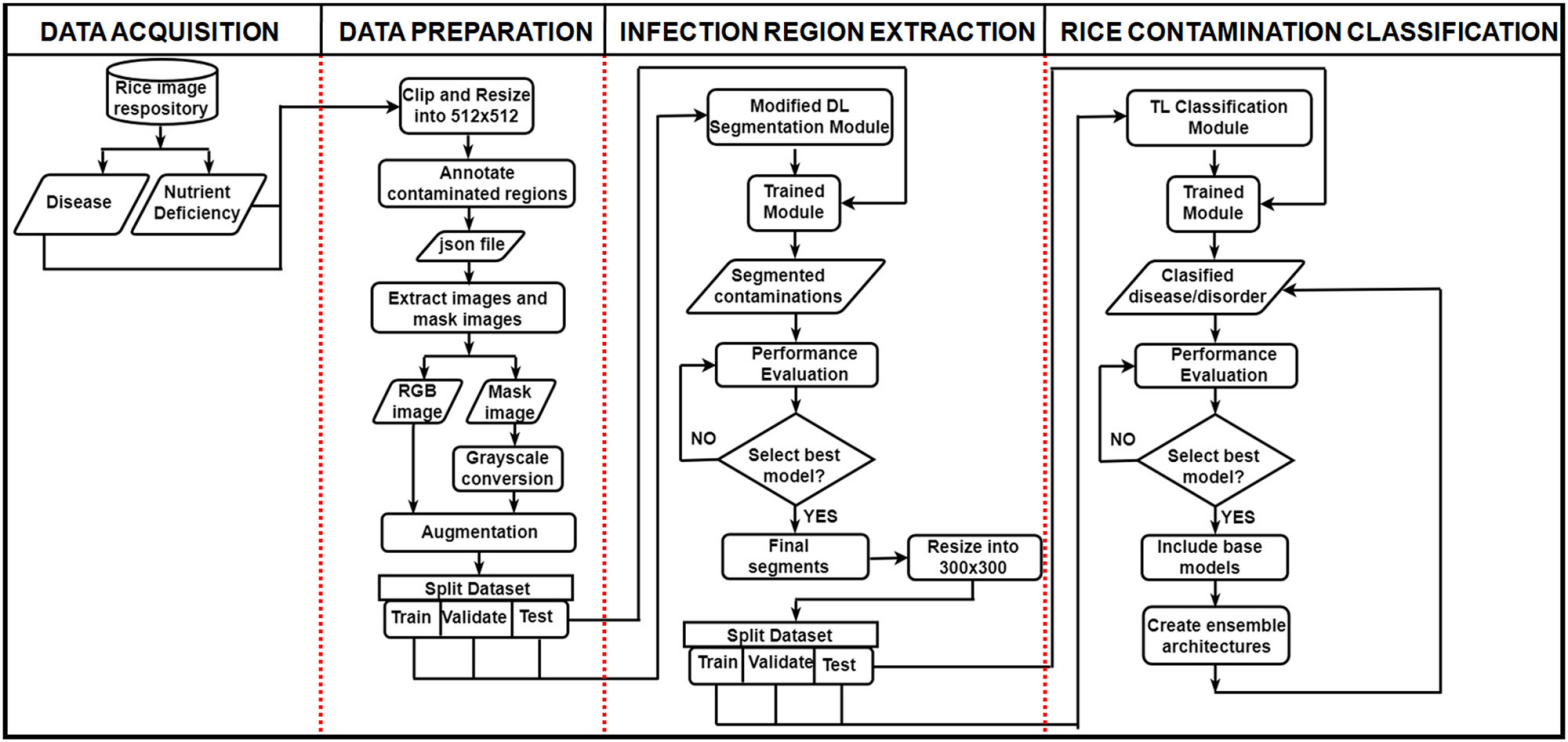
Flowchart of the complete system for disease and deficiency disorder diagnosis in rice leaves.

The DeepBatch presented in this work consists mainly of two modules, and both are designed using various DL architectures. Together, these architectures can enable precise segmentation and classification of rice-infected leaf images. The flowchart of the complete system for rice disease and deficiency disorder diagnosis is shown in Figure 3.

The datasets used in this study were retrieved from the Kaggle repository [38] and UCI ML [41] repository. The images of the input rice disease and deficiency disorders were resized and enhanced. In order to label the infected areas of the images, ground truth masks were created. After creating annotations and extracting masks of the rice leaf images, a dataset was created in the ratio of 70:30 for performing the segmentation of rice diseases. Then DL segmentation architectures were applied to this newly created dataset. The segmentation method was assessed using the following metrics: dice loss, dice coefficient, accuracy, precision, and recall.

The infected portion, omitting the background and the healthy leaf region, was obtained by bitwise multiplying the RGB images and their corresponding predicted masks. Once the segmentation was completed, the properly segmented images were considered for the classification process. The 60 and 20% occurrences of the outputs of the bitwise multiplication were used for training and validation for each classification algorithm, respectively. The remaining 20% of the instances were utilized during testing. TL architectural ensembles (binary, ternary, quaternary, and pentanary) were also proposed, and their results include the infected region’s class and the classifiers’ performance scores.

### 2.1 Data preparation

#### 2.1.1 Image acquisition

We selected two datasets that were sizable enough to offer useful information but not so large that it becomes difficult to annotate contaminated areas. The datasets include the relevant nutrient deficiencies like N, P, and K as well as rice diseases, including BLB, BS, and LSm. Moreover, we were looking for a dataset that could be easily accessed, either through a public repository or by directly contacting the owner of the data. Therefore, the Kaggle and UCI ML datasets were chosen.

As far as law or ethics are concerned, the selected datasets may be used for research purposes without restriction. The acquired dataset contains 120 diseased rice leaf images, which include BLB, BS, and LSm varieties. The images’ resolutions vary from 301 × 71 to 3,081 × 897 pixels. The images were taken in a field in the Shertha village of Gujarat, India, using a 12.3 megapixel NIKON D90 digital SLR camera during the winter season. This dataset has 120 images in the .jpeg format, including 40 images of each disease [41].

**Figure 4:**
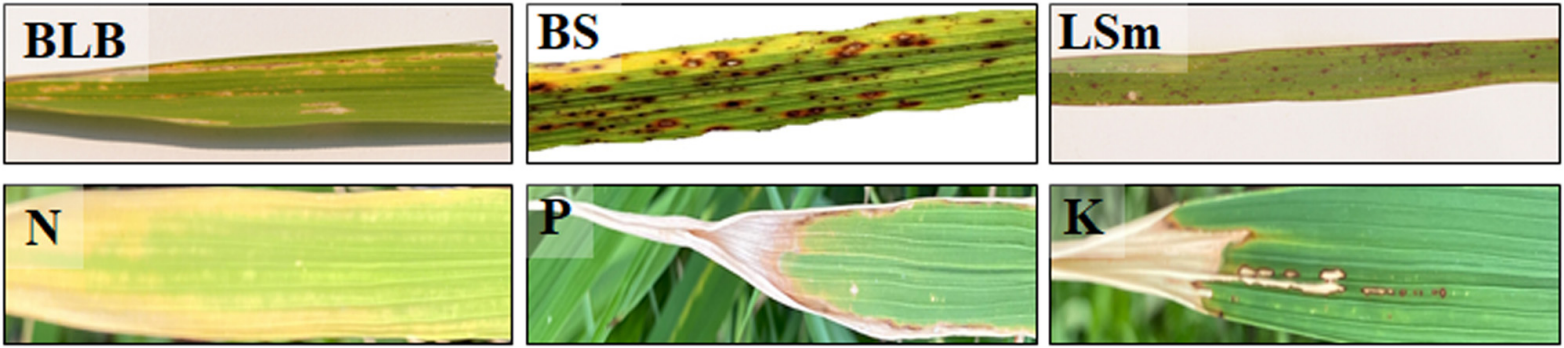
Examples of images from rice diseases and deficiency disorder datasets.

The Kaggle rice nutrient deficiency images consist of three types of deficiencies, namely nitrogen (N), phosphorous (P), and potassium (K). There are 440, 333, and 383 images in each of these classes. All the images are of varying resolutions [8]. A snapshot of the image dataset of rice diseases and deficiency disorders used in this study is shown in Figure 4.

#### 2.1.2 Image pre-processing and rice disease/deficiency disorder annotation

In order to extract multiple disease zones from a single image of a rice leaf, the images of the dataset were clipped. They were then scaled down to 512 × 512 pixels in resolution. After clipping and resizing, a total of 255 rice disease images were obtained. Similarly, 1,156 rice deficiency disorder images were obtained. The clipping and resizing approach used in this study is shown in Algorithm 1. No ground truth segmentation masks were present in the rice datasets. In order to evaluate the task of segmentation, the positions and categories of infected patches in the rice images were labeled using the VIA annotation tool.

**Table:** 

**Algorithm 1**: Algorithm to obtain resized clipped images
**Input**: f1 = path_to_dataset
**Output**: resized_image
For all_images in f1 do
box = clipping_size
image_crop = file_i.crop(box)
resized_image = image_crop.resize((512,512))
resized_image.save(file_i)
End

**Figure 5:**
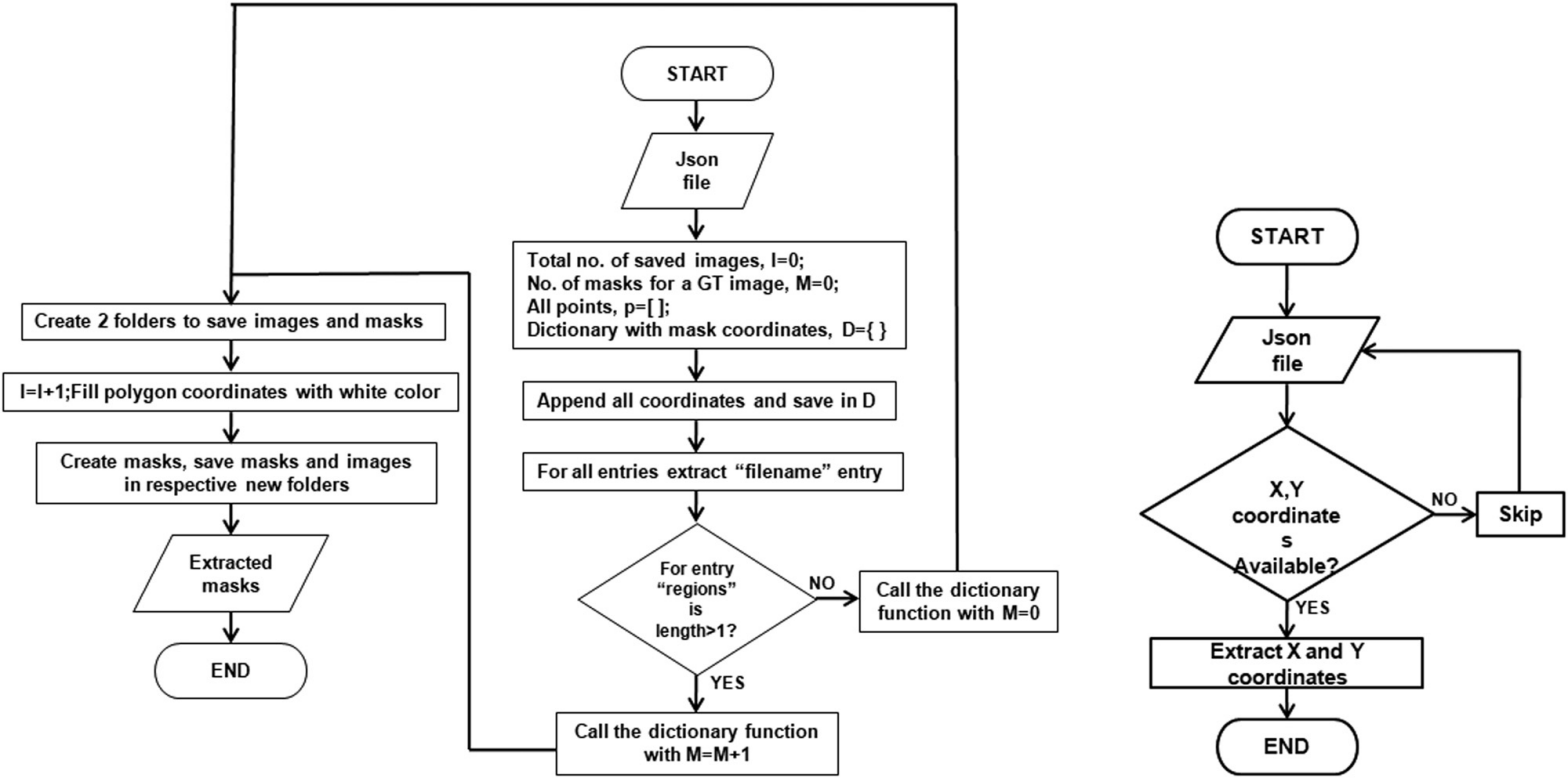
Flowchart of extracting mask images from a labeled json file: (a) main program and (b) save to dictionary function.

The rice-diseased regions were manually refined using a polygonal form. All annotations containing shape and region properties were extracted into a .json file, which contains the name and polygon points along (*x*, *y*) coordinates [42]. The ground truth (mask) images were then obtained from the labeled json file as shown in Figure 5.

These extracted masks were then subjected to grayscale conversion. To increase the data samples, augmentation techniques like rotation, flip, shear, shift, and zoom were applied to the training and validation images and their corresponding masks. The augmentation parameters are given in Table 1.

**Table 1:** Augmentation techniques applied in the current study

Augmentation techniques	Range/mode
Rotation	0.2
Width shift	0.05
Height shift	0.05
Shear	0.05
Zoom	[0.5, 1]
Horizontal flip	Random
Fill	Nearest newly created pixels

### 2.2 Rice-infected region extraction

**Figure 6:**
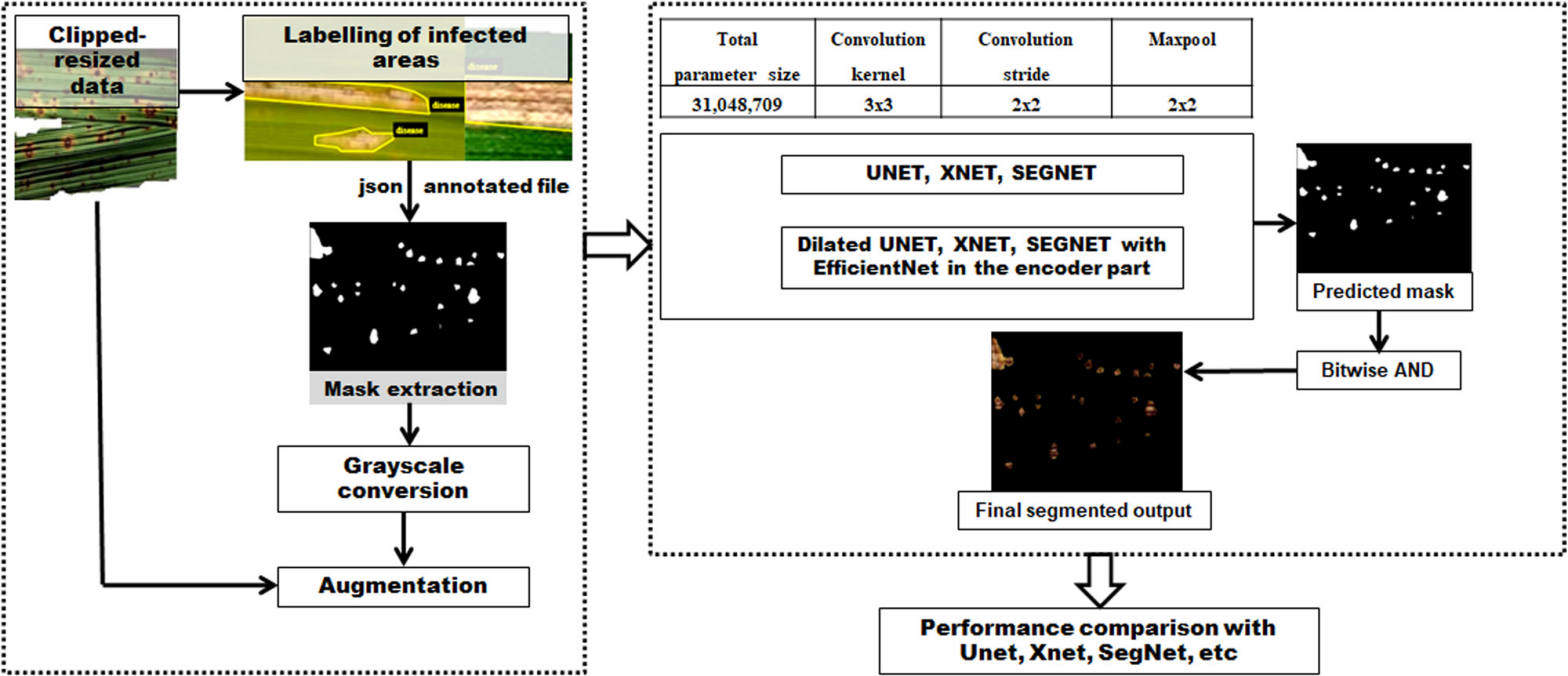
The rice disease DL segmentation framework implemented in this study.

The first module of DeepBatch framework for extracting infected regions from rice images is illustrated in Figure 6. The rice disease and deficiency disorder images were processed separately. These images were divided into training-validation and testing sets in the ratio 70:30, and they were manually labeled to extract the infected regions. The labeled file provided the mask images, which were then converted into grayscale. Augmentation on RGB images and their corresponding grayscale masks resulted in more than 30,000 images and masks, for both datasets. These images were then passed through the DL segmentation models of DeepBatch.

**Figure 7:**
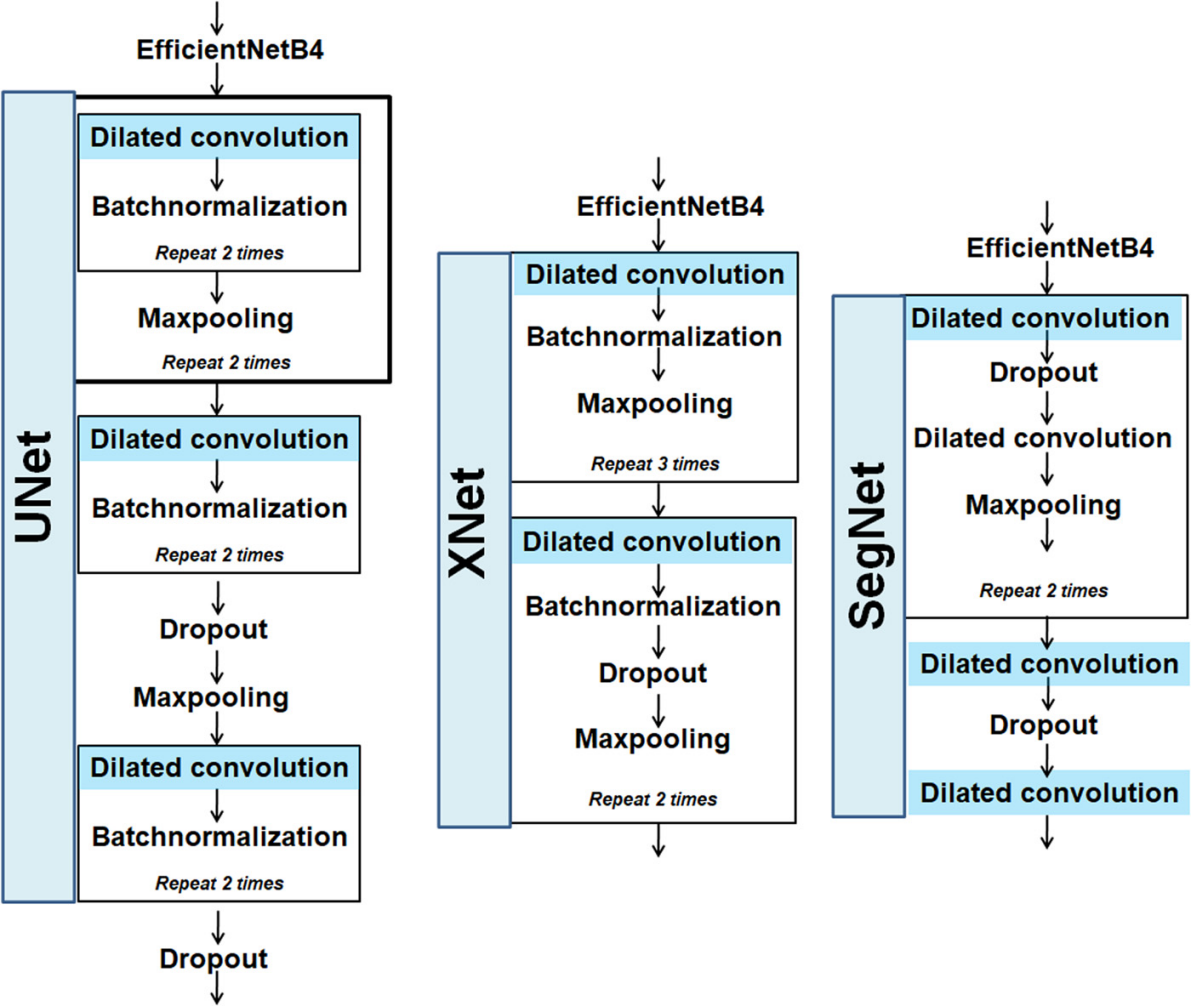
Structure diagrams of encoder blocks of the modified DL segmentation models.

Dilated convolution has been observed to cover maximum input coverage in the classification of images, time series data, sound, etc. [43,44,45]. Therefore, dilated-based enhancements of XNet, SegNet, and UNet-based architectures were introduced for segmenting the infected rice images. In other words, in order to cover more information from the output obtained with every convolution operation, dilation convolution and pre-trained DL model were introduced in the encoder section of the three DL segmentation models. The dropout layers were used to prevent overfitting [46,47,48,49,50]. The modifications done to the existing models are shown in Figure 7. The DL segmentation models were trained for 50 epochs with an adaptive moment estimation (Adam) optimizer whose learning rate was set to 0.001. The dice loss function was used since our datasets contain imbalanced class proportions.

The weights were saved only if there was an improvement in validation loss. The models were executed utilizing GPU with a batch size of 32. The dilation rate used in the present work was *r* = 2. The detailed model layer size used during training is shown in Table 2.

**Table 2:** Layer size of the deep segmentation models used in the current work

Layer	Modified DL segmentation models		
	U-Net	XNet	SegNet
Input	512 × 512 × 3	512 × 512 × 3	512 × 512 × 3
Convolution kernel	3 × 3	3 × 3	3 × 3
Convolution stride	2 × 2	2 × 2	3 × 3
Dilation	2	2	2
Maxpool	2 × 2	2 × 2	2 × 2
Convolution filter	2 × 2	2 × 2	3 × 3
Dropout	0.5	0.5	0.2

#### 2.2.1 UNet and modified UNet

Contracting and extracting pathways are present in U-Net. In the portion of the extracting path, the image size is restored to its original size after being reduced to half in the contracting path. The modified version of U-Net used in the current study has an intermediate layer with two dilated convolutional layers and a dropout of 0.5. The contracting path started with EfficientNetB4 (pre-trained on ImageNet). The image was upsampled and joined with the appropriate image from the contracting path during the extraction process. The upsampling block included convolutional, merging, and upsampling layers. This block was repeated four times before the addition of a convolution layer. The output activation function of the last convolution layer was sigmoid [47,48].

#### 2.2.2 SegNet and modified SegNet

The EfficientNetB4 model was pre-trained on ImageNet data as the first layer of the encoding structure. Its output was received as input by two layers of regularized (0.2) maxpooled convolutions. In the second layer of encoding, regularized convolutions were used. In the decoder, upsampling of convolution layers along with regularized convolutions was applied. The decoder had access to the low-level features created by the encoder layers [46].

#### 2.2.3 XNet and modified XNet

The encoder–decoder-based XNet was observed to be first used in the medical field for X-ray image segmentation. Encoder contains several sequences of convolutional layers for feature extraction and a maxpooling layer to downsample the image. In the modified XNet used in this study, the encoder part of XNet was dilated with a dilation rate of 2 and a dropout probability of 0.5. EfficientNetB4 was included in the encoder path, which was trained on the ImageNet dataset. The convolutional layer followed by maxpooling was repeated three times. Next, layer flattening was performed through two convolution layers. Decoder performed the upsampling to create segmented masks of input BLB images. In the current work, the decoder contained upsampling block which had the upsampling layer and convolutional layer, followed by concatenation. The output of the last activation from the encoder and the output of the present activation of upsampling block were concatenated. This upsampling block was repeated twice after which maxpooled normal convolution and layer flattening were performed. For getting the final segmented mask, the sigmoid activation function was used after three blocks of upsampling [48].

The segments consisted of the predicted binary masks for the infected regions in rice images. The OpenCV image processing library was used to obtain the intersected pixels in both image and its respective predicted mask. The pixels of the RGB images were overlaid over the corresponding predicted masks and a logical bitwise AND operation was performed. The pixels that are common to every image and its mask were considered. The remaining pixels (background and healthy leaf sections) were left out. Hence, the bitwise AND operation would produce the segmented infected region only (in RGB format).

### 2.3 DL rice disease/deficiency disorder classification

The final segments for the rice disease and deficiency disorder were produced by the bitwise AND operation [51]. The properly segmented images were then chosen for feeding into the DL classification system. The resolutions of images were resized to 300 × 300 pixels. Training, testing, and validation sets were created from the images in the following proportions: 60:20:20. The common dataset split of 60:20:20 is a guideline used in the literature [17]. So, we have chosen a split that is suitable for our dataset’s specific needs. The techniques and parameters mentioned in Algorithm 2 were used to augment both datasets. Five TL models, MobileNetV2, InceptionResNetV2, Xception, DenseNet201, and VGG19, were then trained and tested on the datasets. The models were accurately trained using the categorical cross-entropy loss function and the Adam optimizer [5,8].

**Table:** 

**Algorithm 2:** Algorithm to resize and augment images
Input: f1 = Path_to_dataset
Output: final_image
For all_images in f1 do
final_image = file_i.resize(300,300)
final_image.flip_left_right(0.7)
final_image.rotate(0.6, 23, 25)
final_image.skew(0.8, 0.8)
final_image.zoom(probability = 1.0, min_factor = 1.0, max_factor = 1.6)
final_image.sample(403)
End

**Figure 8:**
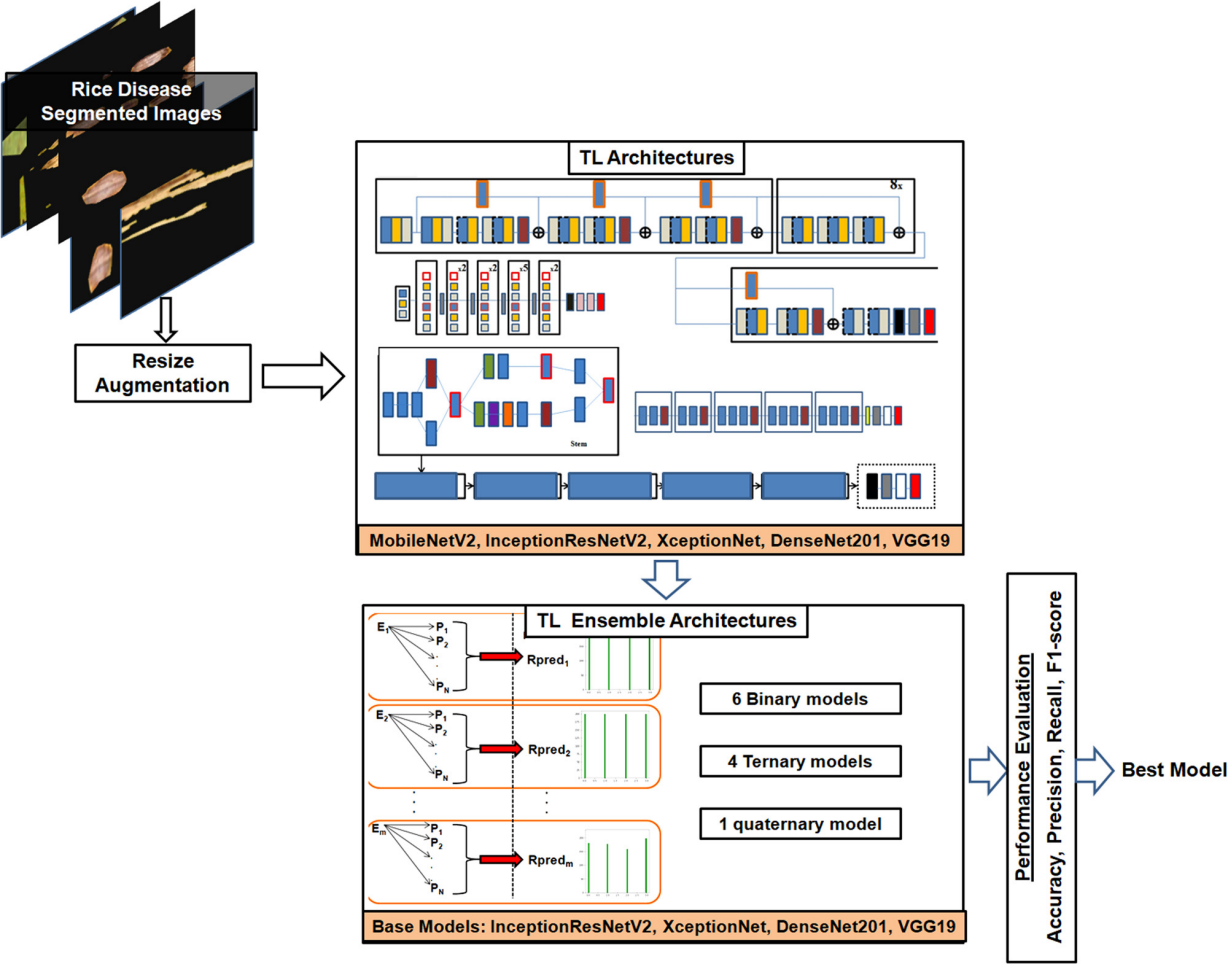
The rice disease DL classification framework implemented in this study.

The highest-performing TL models among them were combined to create ensemble classifiers. All the classifiers were assessed using performance measures such as a confusion matrix, precision, recall, *F*1-score, and accuracy to choose the optimal model for rice disease diagnosis [8,15,16,17]. A comparison was also done on the results of rice disease classification with/without applying the DeepBatch method. The classification module for rice disease diagnosis is shown in Figure 8.

#### 2.3.1 TL architectures

Different industries, including gaming, healthcare, agriculture, autonomous driving, etc., use TL designs extensively. In the current study, the classification of segmented rice disease and deficiency disorders was done using pre-trained models such as VGG19, Xception, DenseNet201, InceptionResNetV2, and MobileNetV2.

InceptionResNetV2 accepts RGB images with 299 × 299 resolution. The first layer consists of a 3 × 3 convolutional layer with 32 filters, a batch normalization layer, and a ReLU activation function. Inception-ResNet-A and Inception-ResNet-B blocks are then stacked after this layer’s output. A reduction block, which combines convolutional and pooling layers, then processes the output.

Following the reduction block, there is a stack of Inception-ResNet-C blocks with various filter sizes. A global average pooling layer processes the output by averaging each feature map across the output’s spatial dimensions. A fully connected layer is then applied to the output of the global average pooling layer, followed by a softmax activation function [5,8]. In this study, InceptionResNetV2’s initial 156 layers are frozen throughout training. The global average pooling layer, dense layer with a ReLU activation, 50% drop out, and dense layer with a softmax activation make up the classification layer.

The NN architecture VGG19 has 19 layers. An RGB image with a resolution of 224 × 224 serves as VGG19’s input. A batch normalization layer and a ReLU activation function are placed after the first layer (a 3 × 3 convolutional layer with 64 filters). The output of this layer is then subjected to a series of 2–4 3 × 3 filter-sized convolutional layers, followed by a max pooling layer. The output is processed by fully connected layers after the convolutional layers and max pooling layers. A final softmax activation function, which generates a probability distribution over the classes, comes after the fully connected layers [5,8,52]. In this study, the last 20 layers of VGG19 are frozen throughout training. The global average pooling layer, a dense layer with a ReLU activation, 50% dropout, and a dense layer with a softmax activation make up the classification layer.

An NN architecture called XceptionNet accepts RGB images up to 299 × 299 pixels as input. A batch normalization layer and a ReLU activation function are placed after the first layer of a 3 × 3 convolutional layer with 32 filters. The next section consists of a stack of convolutional blocks, each of which combines batch normalization layers, ReLU activation functions, and depthwise separable convolutions. The output is processed by a global average pooling layer after the convolutional blocks, which calculates the average of each feature map across the spatial dimensions of the output [5,8,53]. In this study, the last 20 layers of XceptionNet are frozen throughout training. The global average pooling layer, a dense layer with a ReLU activation, 50% dropout, and a dense layer with a softmax activation make up the classification layer.

DenseNet201 accepts RGB images with 224 × 224 resolution as input. The first layer is a 7 × 7 convolutional layer with 64 filters followed by a 3 × 3 maximum pooling layer. A string of dense blocks, each containing a number of convolutional layers with varying filter sizes, is then applied to the output of the pooling layer. A global average pooling layer follows the dense blocks and averages each feature map across the output’s spatial dimensions. A fully connected layer with a number of classes and a softmax activation function follows the output of the global average pooling layer [5,8]. In this study, the last 20 layers of DenseNet201 are frozen throughout training. The global average pooling layer, a dense layer with a ReLU activation, 50% dropout, and a dense layer with a softmax activation make up the classification layer.

An NN architecture called MobileNetV2 accepts RGB images of 224 × 224 as input. There are 32 filters, a batch normalization layer, and a ReLU activation function in the top layer. A batch normalization layer, a depthwise separable convolution, and a ReLU activation function are all present in each of the 16 residual bottleneck blocks that follow this layer. The gradient signal is transmitted from the output to the input by employing skip connections to connect the remaining bottleneck blocks. Following residual bottleneck blocks, the output is processed by a global average pooling layer. The average of each feature map is calculated across the output spatial dimensions by this layer. A feature vector is generated by this layer, and it will be used for the targeted task [5,8,52]. In this study, the last 20 layers of MobileNetV2 are frozen throughout training.

These models were trained for 50 epochs with a batch size of 32 to ensure that proper learning takes place. The optimal number of epochs for each TL model was tracked using the early stopping method. Overfitting was avoided by a 50% dropout rate.

#### 2.3.2 TL ensemble architectures

**Figure 9:**
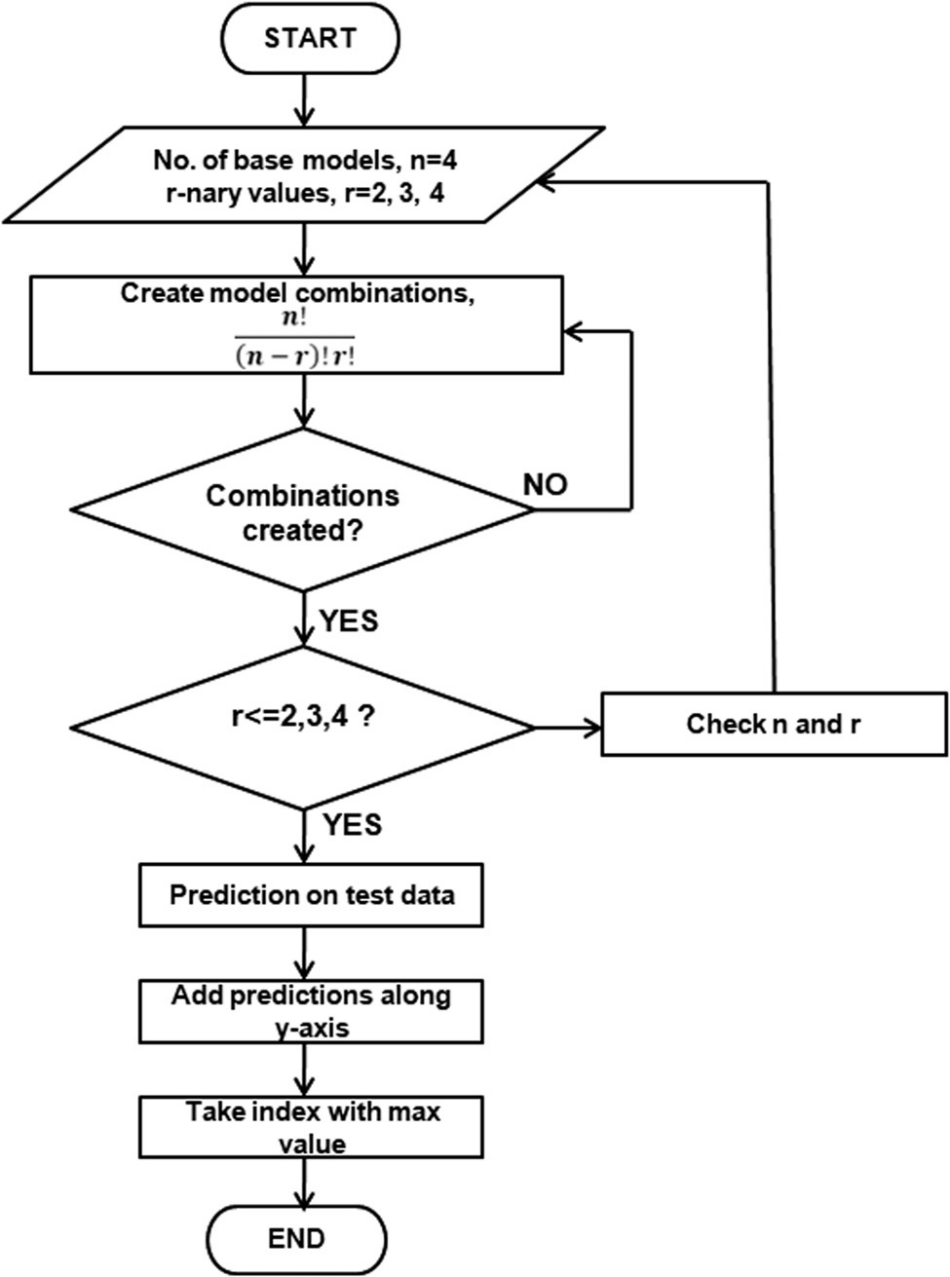
Flowchart of TL ensemble averaging implemented in this study.

The four base TL models’ predicted probabilities were combined in the current study using ensemble averaging. These four base learners were used to create various binary, ternary, and quaternary ensemble classifier combinations. As shown in Figure 9, the test set of the dataset was used to calculate the prediction probabilities generated by each combination of classifiers.

### 2.4 Performance assessment

The metrics used for analyzing DL segmentation were dice coefficient, dice loss, precision, and recall. In the classification part of DeepBatch, precision, recall, *F*1-score, and accuracy were used to evaluate each classification model.

#### 2.4.1 Dice coefficient

The Dice coefficient, a similarity statistic, is utilized to evaluate the effectiveness of image segmentation models. For determining the Dice coefficient, the predicted and ground truth masks of a binary image are compared.

Masks are binary images that contain only the ROIs and set everything else to zero. Dice coefficients are used to determine the accuracy of segmentation in the model. These coefficients determine the overlap between the predicted masks and the actual masks. Dice coefficients, which range from 0 to 1, determine whether the predicted and ground truth masks completely overlap or not. The dice coefficient is calculated as the ratio of the twice-intersection area and the total area between the input mask and the predicted mask [43,54].

#### 2.4.2 Dice loss

ML models for image segmentation are trained using dice loss functions. It is derived from the Dice coefficient, a similarity statistic used to evaluate image segmentation performance. A dice loss is a quantitative measure of the difference between a predicted mask and an actual mask based on the Dice coefficient. Calculated as 1 minus the Dice coefficient, the dice loss represents the degree of similarity between the predicted and actual masks. Therefore, the objective of the model is to minimize the Dice loss by increasing the Dice coefficient and maximizing segmentation accuracy [43,54].

#### 2.4.3 Recall and precision

Performance measures such as recall and precision are used to assess the accuracy of a classification model. To generate both metrics, a confusion matrix is used, which lists the total number of true positives, false positives, true negatives, and false negatives. Referred to as sensitivity, recall measures the percentage of accurate positive predictions among all real positive cases. A high recall score indicates that a significant portion of the positive examples was accurately predicted by the model. A high recall score, for instance, indicates that the model correctly detects the majority of diseases in agricultural crops. Precision is the percentage of correctly predicted classes that are positive cases. A high precision score suggests that the model is producing fewer false-positive predictions. For example, of all crops predicted to have blight disease, the precision score gives us the number of crops actually infected with blight [5,8].

#### 2.4.4 *F*1-score and accuracy

ML uses *F*1-score and accuracy to evaluate the effectiveness of classification models. The confusion matrix, a table that lists the total number of true positives, false positives, true negatives, and false negatives, is used to calculate them. Accuracy is defined as the ratio of correct predictions made by the model to total predictions. Accuracy is beneficial in situations where there are roughly equal number of positive and negative cases. When classes are unbalanced, it may be difficult to measure accuracy. For example, in crop disease diagnosis, the model may have a high accuracy score, yet be useless as a diagnostic tool if it predicts that all crops are negative [5,8,40,52,55,56,57,58,59].

## 3 Results

The training and the validation of various learning models were carried out using GPU available in the Google Colab platform. Sections 3.1 and 3.2 explain the results obtained from the two stages of DeepBatch considered in this study.

### 3.1 Results of module 1 of DeepBatch

The DL segmentation framework using the modified versions of U-Net, XNet, and SegNet was used to extract infected regions in 255 rice leaf disease images and 1,156 rice deficiency disorder images. Considering the results of extracting regions infected with rice diseases, it was observed that the optimal loss value of the modified UNet segmentation changed from 0.8 to 0.22 after 50 epochs. Similarly, the optimal loss value of the modified SegNet segmentation changed from 0.86 to 0.26.

From Table 3, it was observed that Dilated U-EfficientNet performed better than the rest in terms of dice loss, dice coefficient, and recall. Both UNet and dilated Seg-EfficienNet had validation losses of 0.8 after 50 epochs, which were determined to be worse than those of the other models. So, in order to conduct further comparisons, these segmentation models were ignored.

**Table 3:** Quantitative evaluation for extracting diseased regions in the rice leaf (module 1)

Models	Dice loss	Dice coefficient	Precision	Recall	Accuracy
UNet	0.8584	0.1416	0.7587	0.8947	0.9828
XNet	0.2267	0.7733	0.8723	0.7358	0.9840
SegNet	0.3588	0.6412	0.5364	0.8691	0.9600
Modified SegNet	0.8636	0.1364	0.0769	0.7905	0.5801
Modified XNet	0.2241	0.7759	0.8600	0.7564	0.9846
Modified UNet	0.2234	0.7766	0.8876	0.7112	0.9836

**Figure 10:**
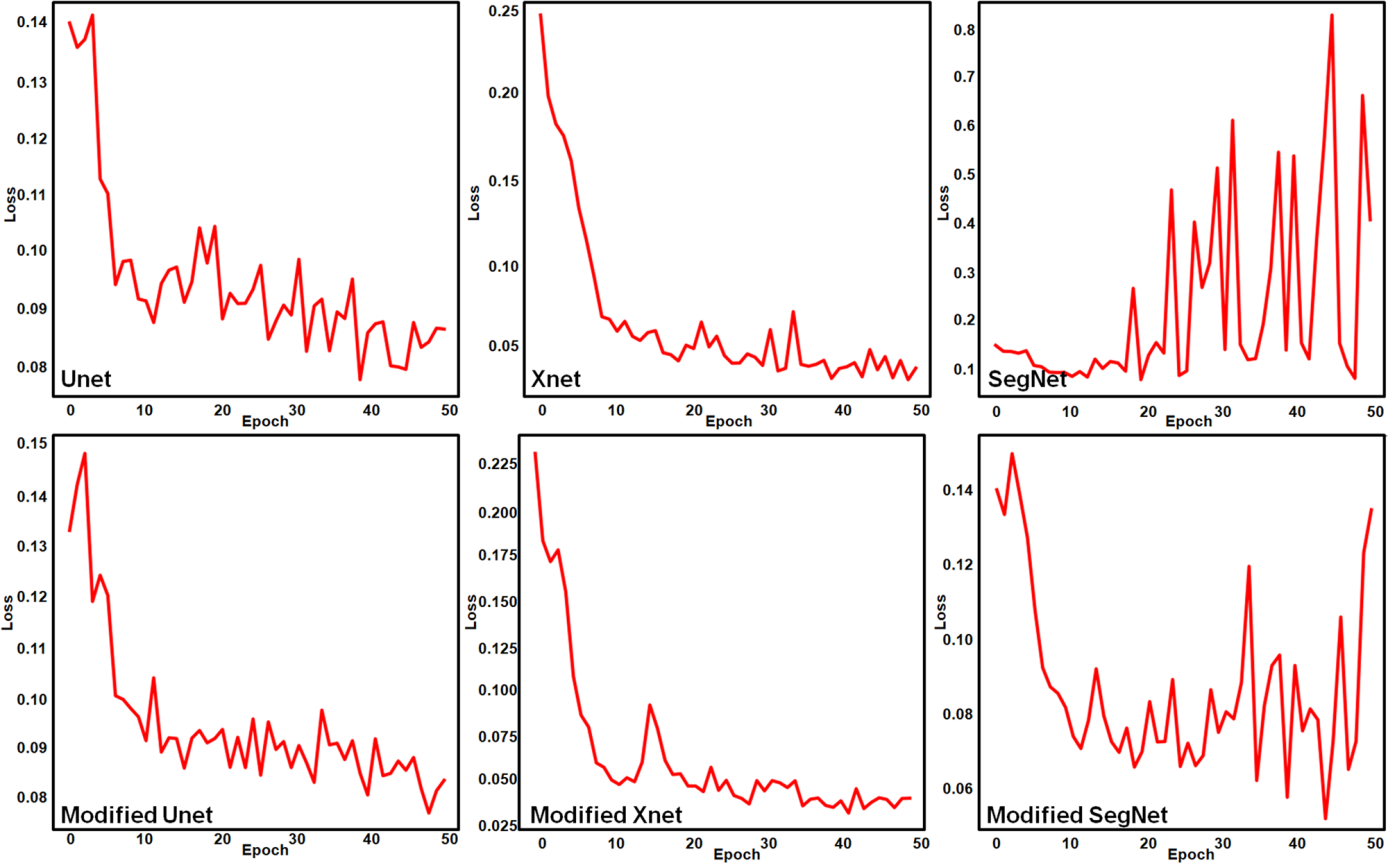
Validation loss curves during the extraction of rice-diseased regions using DL segmentation.

In the remaining models, a pattern of validation loss across 50 epochs was seen. Figure 10 shows the loss function curves while extracting rice disease regions using DL segmentation models. It was found that although the validation loss for XNet, Dilated X-EfficientNet, and Dilated U-EfficientNet after 50 epochs was found to be 0.2, frequent fluctuations were seen in the cases of XNet and Dilated X-EfficientNet. As a result, Dilated U-EfficientNet and SegNet-EfficientNet, with a precision of 88.76 and 88.65%, respectively, were identified as the best models to obtain segmented regions of rice diseases.

**Figure 11:**
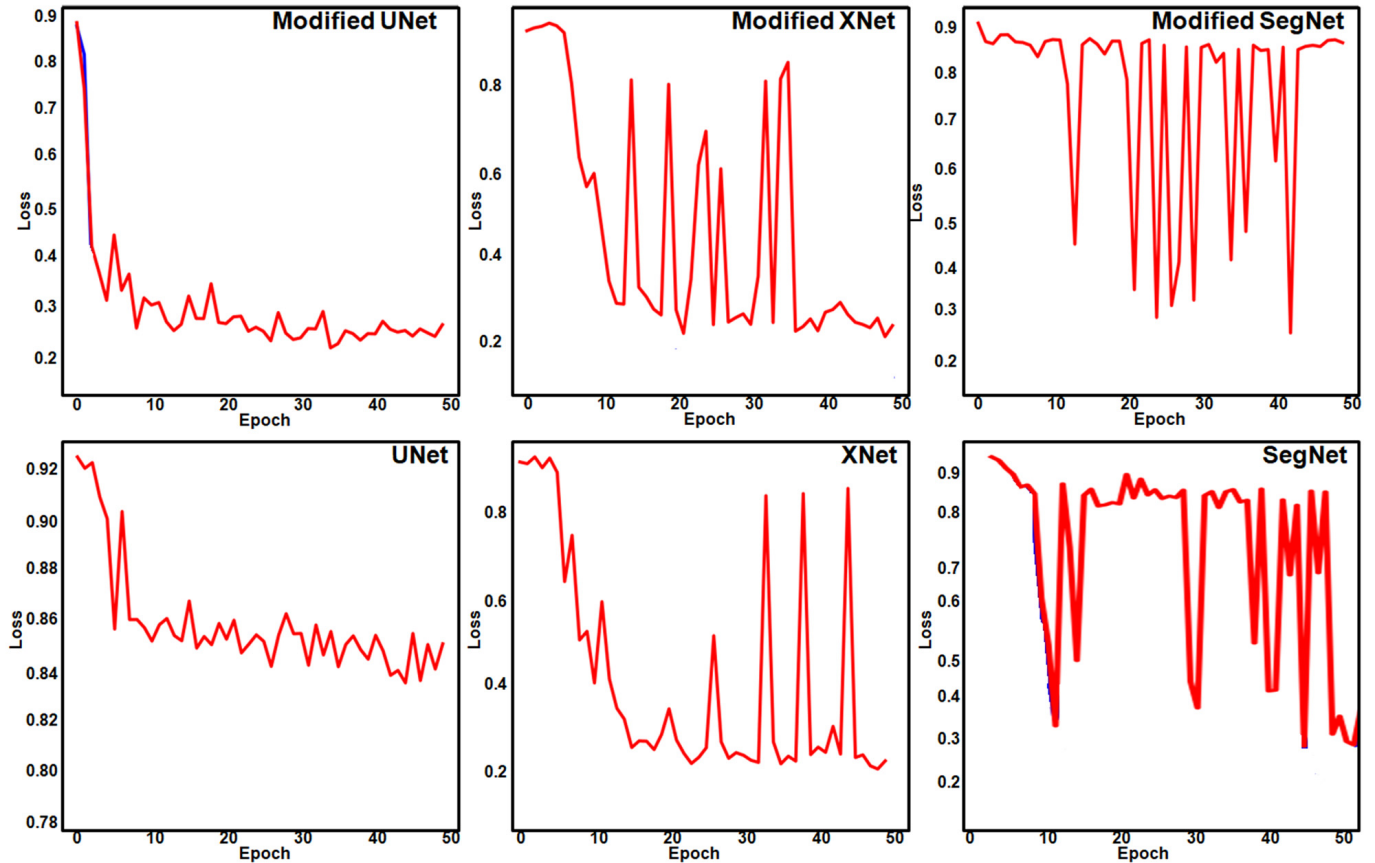
Validation loss curves during the extraction of rice deficiency disorder regions using DL segmentation.

Figure 11 shows the behavior of the DL architectures for extracting regions with rice nutrient deficiency disorders. Table 4 shows the quantitative results of extracting deficiency disorder regions. It was found that although the optimal loss for SegNet and Dilated U-EfficientNet after 50 epochs was found to be between 0.2 and 0.4, frequent fluctuations were observed. Hence, these models were ignored along with the modified SegNet. The values of the loss function at each epoch showed that all the models except XNet and modified XNet suffered from very high overfitting. This overfitting got reduced to a large extent in XNet and modified XNet.

**Table 4:** Quantitative results of extracting rice deficiency disorder regions using DL segmentation (module 1)

Models	Dice loss	Dice coefficient	Precision	Recall
UNet	0.0866	0.9134	0.9702	0.9673
XNet	0.0373	0.9627	0.9587	0.9717
SegNet	0.4034	0.5966	0.9913	0.4535
Modified UNet	0.0820	0.9180	0.9747	0.9697
Modified XNet	0.0363	0.9638	0.9490	0.9829
Modified SegNet	0.8636	0.1364	0.0769	0.7905

The average loss in the latter was decreased by 0.0004, which was considered the best model of the six DL models. The optimal loss value of the modified XNet segmentation model changed from 0.2310 to 0.0362 throughout 50 epochs.

**Figure 12:**
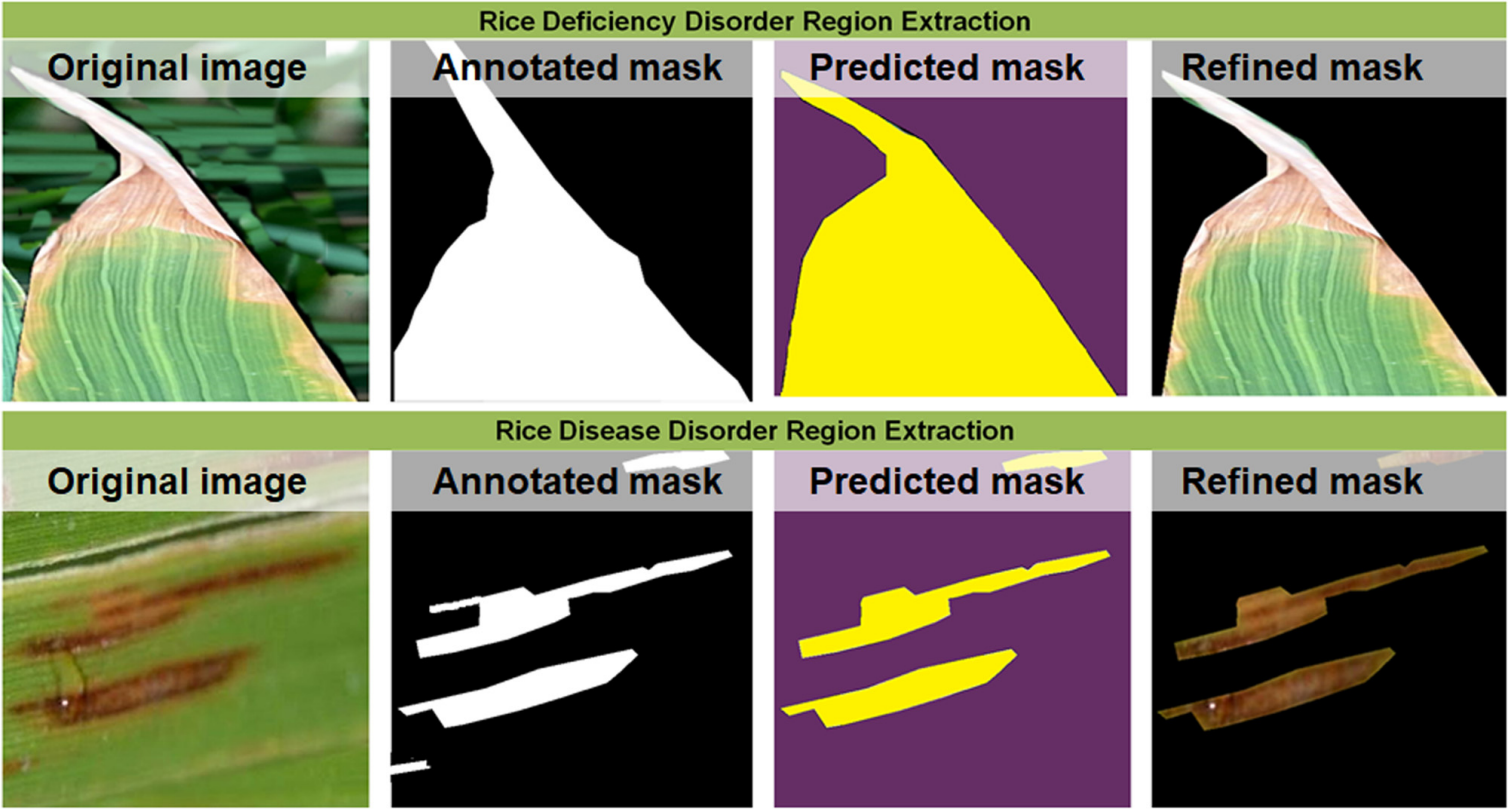
Extraction of the rice-infected region showing the input images, human annotation, mask produced by DL infected region extraction framework, and the refined segmented region.

The qualitative results for the DL segmentation of infected rice leaves (disease and deficiency disorders) in the rice-infected region extraction module of the DeepBatch framework are shown in Figure 12, where the white/yellow color indicates an infected region. In contrast, the black/purple color indicates a non-infected region. According to qualitative evaluation, a relative similarity is observed between the regions labeled by humans and those inferred by the best DL segmentation architecture. The refined segment obtained by applying bitwise logical AND operation on the images and their predicted masks is given attention in the next stage of DeepBatch. Finally, the predicted masks of rice diseases produced by dilated U-EfficientNet were considered as they showed the best performance. The predicted masks along with their respective RGB images underwent bitwise AND operation. In comparison with the human annotations, the ability of the best DL segmentation architecture to separate background pixels within infected regions is shown by the dice coefficient curves.

**Figure 13:**
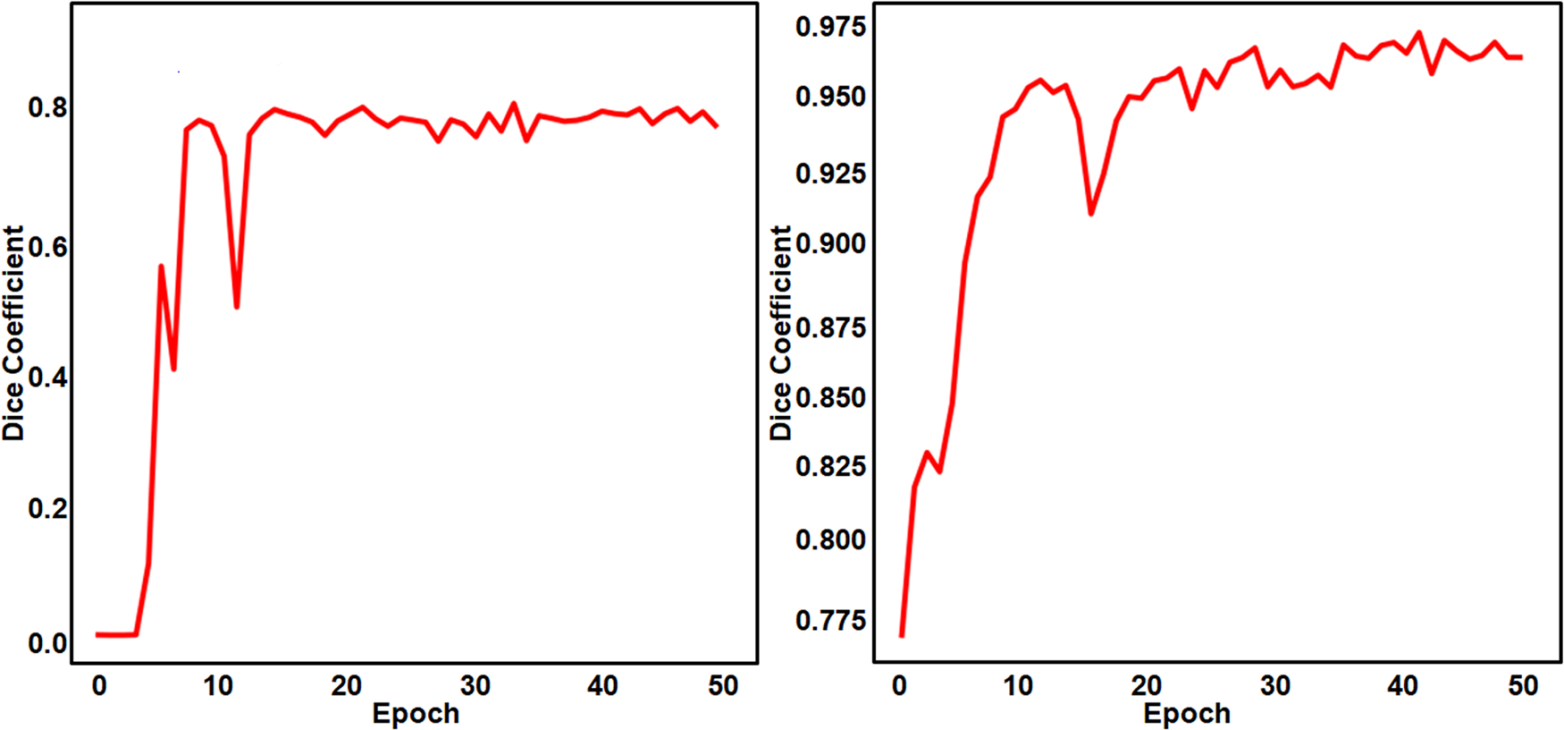
Dice coefficients for segmenting (a) rice diseases and (b) rice deficiency disorders.

It was seen that the optimal dice value of the segmentation results reached 0.77 and 0.96 while extracting regions infected by disease and deficiency disorders, respectively, in rice leaves. Figure 13 shows the dice curves of DeepBatch module 1.

### 3.2 Results of module 2 of DeepBatch

The Google Colab platform’s GPU was used to perform the training and validation of several learning models for classifying the segmented regions using DeepBatch. The segmented rice-infected images obtained from DeepBatch module 1 were processed using five TL models, i.e., MobileNetV2, InceptionResNetV2, Xception, DenseNet201, and VGG19. The ensemble versions of the top TL models which were considered are shown in Table 5 along with their performance measures. Considering the rice disease dataset, the accuracy scores of all these models were in the range of 49.67–94.33. The performance of InceptionResNetV2 with 94.33% accuracy was the highest as compared to other TL models.

**Table 5:** Quantitative results of rice disease and deficiency disorder classification after applying DeepBatch showing precision (*P*), recall (*R*), *F*1, and accuracy (*A*) scores

Models	Rice disease				Rice nutrient deficiency disorder			
	*P*	*R*	*F*1	*A*	*P*	*R*	*F*1	*A*
TL0: InceptionResNetV2	94.33	94.33	94.33	94.21	93.67	92.67	92.67	92.67
TL1: VGG19	92.33	92.33	92	92.16	86	83	82	83
TL2: XceptionNet	93.33	93	93	93.31	85.67	78	77.33	78
TL3: DenseNet201	94	93	93.33	93.22	88	85	85	85
TL4: MobileNetV2	79	53.67	49.67	53.59	77	48.67	44.33	48.67
EM2.0: InceptionResNetV2 + DenseNet201	93.33	96	96	96.20	94	93.33	93.33	93.33
EM2.1: InceptionResNetV2 + VGG19	94.67	94.67	94.67	94.47	92.67	92	92	92
EM2.2: InceptionResNetV2 + XceptionNet	95.67	95.67	95.67	95.62	94	93.33	93.33	93.33
EM2.3: XceptionNet + VGG19	95	94.67	95	94.72	89.67	88	87.67	88
EM2.4: DenseNet201 + VGG19	95.67	95.67	96	95.70	86.33	85.33	85	85.33
EM2.5: DenseNet201 + XceptionNet	94.67	95	94.67	94.79	91.33	89.33	89.67	89.33
EM3.0: EM2.2 + VGG19	96.33	96.33	96.33	96.28	92.33	91.33	91	91.33
EM3.1: EM2.2 + DenseNet201	97	96.67	97	96.94	94.67	94	93.67	94
EM3.2: EM2.3 + DenseNet201	96	95.67	96	95.62	90.67	90	89.67	90
EM3.3: EM2.5 + DenseNet201	96	96.33	96.33	96.20	90.67	90	89.67	90
EM4.0: EM2.2 + VGG19 + DenseNet201	96.67	96.67	96.67	96.61	93.33	92.67	92.33	92.67

**Figure 14:**
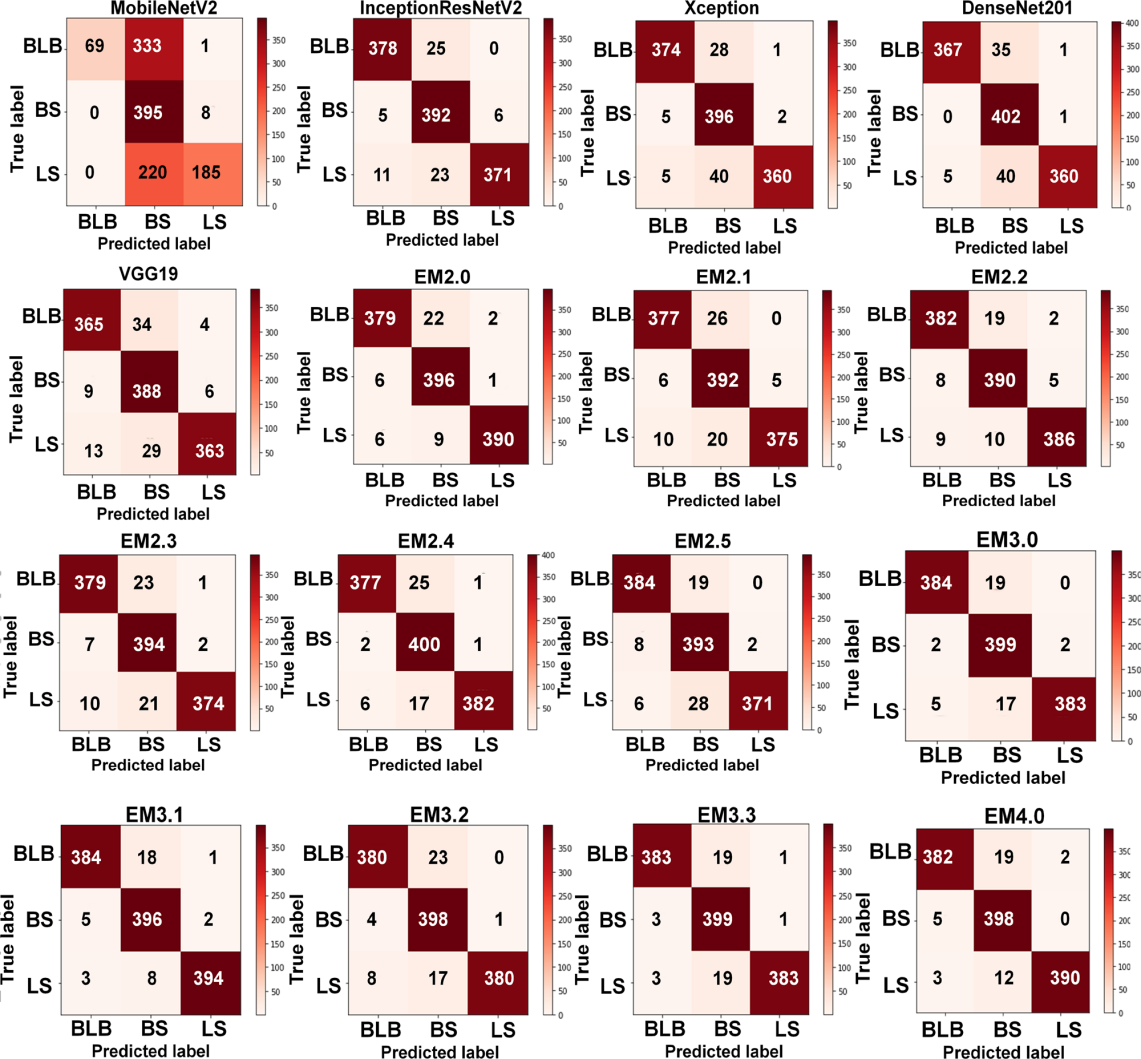
Confusion matrix of all TL and ensemble learning models for classifying the segmented disease in rice.

Figure 14 presents the confusion matrices of the TL and ensemble methods for the segmented input, which shows both the classification and misclassification information. Using the confusion matrices, it has been noticed that BLB and BS showed the least and highest diagnostic accuracies, respectively. MobileNet misclassified BLB and LS in most cases. Only 1 BS sample and 7 samples of BS were misdiagnosed as BLB or LS by DenseNet201 and Xception, respectively. For the remaining models, InceptionResNetV2 and VGG19, BS was more properly classified than BLB and BS.

According to Table 5, the classification accuracies of binary and ternary ensemble TL classifiers for the segmented rice disease images ranged from 94.47 to 96.2% and 95.62 to 96.94%, respectively, with EM3.1 outperforming the competition with an accuracy of 96.94%.

With an accuracy of 94.47%, EM2.1 underperformed all other binary and ternary ensemble TL models. EM2.1 and EM2.4 identified less number of BS samples (377 of 403 samples). Of all the binary ensemble models, the highest number of BLB, BS, and LS samples was classified by EM2.5, EM2.4, and EM2.0, respectively. Of the ternary models, the highest number of BS samples was classified by EM3.0 and EM3.3. Similarly, EM3.0 and EM3.1 correctly classified the BLB samples in most of the cases, whereas the highest number of 394 LS samples was classified by EM3.1. The accuracy of the quaternary ensemble model was 96.61%.

**Figure 15:**
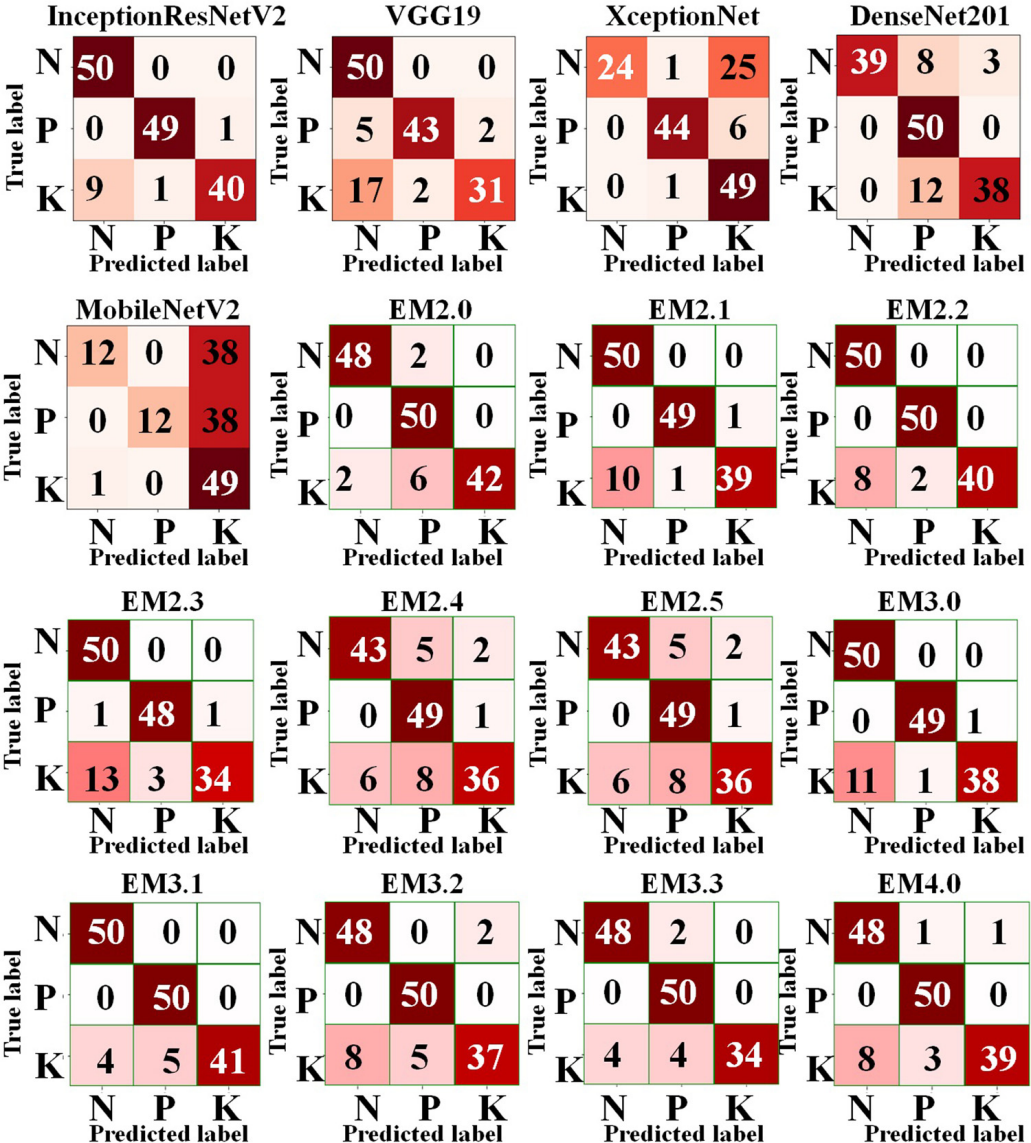
Confusion matrix of all TL models for classifying the segmented deficiency disorders in rice.

Considering the rice deficiency disorder dataset, the accuracy scores of all TL models were in the range of 48.67–92.67%. The performance of InceptionResNetV2 with 92.67% accuracy was the highest as compared to other TL models. Figure 15 presents the confusion matrices of the TL and ensemble methods for the segmented rice deficiency disorders, which shows both the classification and misclassification information. Using the confusion matrices, it has been noticed that P and K showed overall good diagnostic accuracy in all the TL models. InceptionResNetV2 and VGG19 classified all N samples correctly, whereas N was misclassified in most of the cases by MobileNetV2 and XceptionNet. Although 11 N samples and 12 K samples were misclassified by DenseNet201, all P samples were correctly diagnosed. According to Table 5, the classification accuracies of binary and ternary ensemble TL classifiers for the segmented rice deficiency disorder images ranged from 85.33 to 93.33% and 90 to 94%, respectively, with EM3.2 outperforming the competition with an accuracy of 94%.

With an accuracy of 85.33%, EM2.5 underperformed all other binary and ternary ensemble TL models. The binary and ternary ensemble models were able to identify the N and P samples in most of the cases, Of the binary ensembling models, the highest number of N samples was classified by EM2.1, EM2.2, and EM2.3, whereas the highest number of P samples was classified by EM 2.0 and EM2.2. EM 2.0 had also classified the highest number of K samples. Of the ternary models, the highest number of N samples was classified by EM3.0 and EM3.1. Similarly, EM3.1, EM3.2, and EM3.3 correctly classified the P samples, whereas the highest number of 41 K samples was classified by EM3.1.

## 4 Discussion

The use of ML to assist agricultural experts has been widely explored using publicly available/on-field datasets. However, these approaches suffer from a lack of effective features and make it challenging to apply in real environments. Some studies [41,60] have used image processing, segmentation techniques, and data mining to separate the infected portion from rice leaves and applied SVM with a Gaussian kernel to classify these diseases. The results showed that more effective features need to be extracted for ML rice disease classification. As a result, DL algorithms have been favored by most researchers nowadays to independently extract features. TL architectures were found to work better than SVM and CNNs because they prevent the model from becoming saturated with huge data or deeper networks [61]. The ensemble of TL architectures enhanced the accuracy of diagnosing rice disease and deficiency disorders [5,8,62]. However, the goal of the present study is to enhance it further by using a novel framework based on DL algorithms. As compared to other ML/DL methodologies, the present framework has the ability to analyze more features during the diagnostic process and extract critical information from images of both disease and deficiency disorders in rice. The detection of infected regions is based on masks provided by researchers and ensures maximum overlapping between predicted masks and ground truth masks. A direct quantitative comparison with all the related works is of less relevance due to the different datasets and objectives used in these studies.

Tables 6 and 7 organize a quantitative comparison with related works that have used the same datasets.

**Table 6:** Comparison of the DeepBatch model with the related works using the same rice disease dataset

Study	Objective	Rice dataset	Best performance (%)	
			DC	*A*
Kanuparthi et al. [62]	Classification of rice diseases by a ternary ensemble of TL models	UCI BLB, BS, and LSm diseases	_	96.42
Das and Sengupta [60]	Rice disease detection and classification using image processing and data mining	UCI BLB, BS, and LSm diseases	_	95
Patidar et al. [61]	Detection and classification of rice diseases using Deep ResNet	UCI BLB, BS, and LSm diseases	_	95.83
Prajapati et al. [41]	Detection and classification of rice diseases using global feature + SVM (Gaussian kernel)	UCI BLB, BS, and LSm diseases	_	73.33
DeepBatch (present work)	Segmentation of infected rice regions with a pre-trained model and dilated convolution in encoder Classification of segments by ensemble of TL models	UCI BLB, BS, and LSm diseases	77.66	96.94

**Table 7:** Comparison of the DeepBatch model with the related works using the same rice deficiency dataset

Study	Objective	Rice dataset	Best performance (%)	
			DC	*A*
Sharma et al. [8]	Classification of rice NPK deficiencies using an ensemble of TL models	Kaggle NPK deficiency	_	91.33
DeepBatch (present work)	Segmentation of infected rice regions with a pre-trained model and dilated convolution in encoder Classification of segments by ensemble of TL models	Kaggle NPK deficiency	96.38	94

Considering the rice disease dataset, it is inferred from module 1 of DeepBatch that UNet performed the worst segmentation of rice disease regions with a loss of 0.8584. The low loss and high accuracy in modified UNet showed the ideal case of generating very less errors in creating rice disease masks. According to module 2 of DeepBatch, the individual TL, binary, ternary, and quaternary ensemble classifiers had average accuracies of 85.29, 95.25, 96.26, and 96.61%, respectively. The quaternary ensemble model EM4.0 showed 96.61% accuracy [63,64,65].

The classification accuracy of the ensemble TL models for the entire experiment was in the range of 94.47–96.94%, whereas individual TL [66–69] models showed an accuracy of 53.59–94.21%, which confirms the superiority of the ensemble approach [60,70–73]. On applying the DeepBatch method, the validation accuracies of the TL models increased after 50 epochs. Average accuracies for the DeepBatch rice disease diagnosis employing TL models and ensemble models were 85.29 and 95.74%, respectively. DeepBatch has increased the efficiency of TL models and ensemble models by 6.098 and 1.99%, respectively.

Considering rice deficiency disorders, it is inferred from module 1 of DeepBatch that modified XNet performed the best in segmenting regions infected with rice deficiency disorders. In module 2 of DeepBatch, it is observed that the individual TL, binary, and ternary ensemble classifiers [74–78] had average accuracies of 77.468, 90.22, and 91.33%, respectively. The quaternary ensemble model EM4.0 showed 92.67% accuracy. The classification accuracy of the ensemble TL models for the entire experiment was in the range of 85.33–94%, whereas individual TL models showed an accuracy of 48.67–92.67%, which confirms the superiority of the ensemble approach. It is clear from Table 8 that DeepBatch has increased the efficiency of diagnosing both disease and deficiency disorders in rice images. Thus, the proposed method is best fit for rice disease/deficiency disorders diagnosis as it is based on a fusion of segmentation and classification approach using various ensemble of TL models [79–82].

**Table 8:** Mean accuracies (in %) of the best TL models and their ensembles

	Without DeepBatch		With DeepBatch	
Models	Rice disease	Rice NPK deficiency disorder	Rice disease	Rice NPK deficiency disorder
TL models	79.2	82.92	93.23	84.67
Ensemble models	93.75	88.69	95.74	90.85

It is important to be aware of the limitations and biases of this study. In this study, only a few rice diseases and deficiency disorders were considered. All diseases and nutrient deficiencies must be considered for the system to be appropriate for real-world implementation. Additionally, this study only took a few DL segmentation models into account [83–85]. The performance of other DL models, such as variants of UNet, is not investigated in this work. In addition, the datasets considered are benchmark data collected from a particular region [86–88]. Therefore, the experimental result obtained may not be generalized to rice variants, which are being produced in other parts of the world. Furthermore, the study relies only on the image data for the diagnosis, ignoring the fact that multiple other symptoms could have been missed out by image data.

## 5 Conclusion

The DeepBatch methodology proposed in the current study makes use of DL-based segmentation and DL-based classification frameworks. The DL segmentation framework receives pre-processed images of the rice disease together with the corresponding masks. In order to predict the masks of the infected rice parts, the DL segmentation framework used modified versions of UNet, XNet, and SegNet. Bitwise multiplication was used to extract only the contaminated segment, leaving out the background and healthy sections. The DL classification framework receives these improved segmented images as inputs. For identifying diseases in rice plants, the second framework included both ensemble and TL architectures, which were carried out using five TL architectures: InceptionResNetV2, VGG19, Xception, DenseNet201, and MobileNet. The use of Xception, DenseNet201, InceptionResNetV2, and VGG19 produced 11 ensemble models. When compared to individual TL models, the ensembled models’ performance was significantly better. Overall, it is observed that after applying the DeepBatch approach, the average accuracy of the TL models increased in both rice diseases and deficiency disorder images. The experiment demonstrates how the combination of various DL frameworks may be useful to design a rice disease/deficiency disorder diagnostic system. Future studies may concentrate on identifying disease and nutrient deficiency disorders in rice from a larger study region.
